# DEMONSTRATION OF COMMUNITY-ENGAGED RESEARCH CONTINUUM THROUGH A UNIVERSITY AND AREA AGENCY ON AGING PARTNERSHIP

**DOI:** 10.1093/geroni/igae098.0647

**Published:** 2024-12-31

**Authors:** Marisa Sheldon, Katie Calhoun, Smitha Rao, Christine Happel, Holly Dabelko-Schoeny, Anthony Traver

**Affiliations:** The Ohio State University, Age-Friendly Innovation Center, Columbus, Ohio, United States; The Ohio State University, Columbus, Ohio, United States; The Ohio State University, Columbus, Ohio, United States; Age-Friendly Innovation Center, The Ohio State University, College of Social Work, Columbus, Ohio, United States; The Ohio State University, Columbus, Ohio, United States; The Ohio State University, Columbus, Ohio, United States

## Abstract

Area Agencies on Aging (AAA), which were originally established to support the planning and delivery of community-based support, are well-positioned to build knowledge and develop innovative responses to community needs through community-engaged research partnerships. This presentation illustrates a relationship between the Age-Friendly Innovation Center (AFIC) at The Ohio State University College of Social Work and the Central Ohio Area Agency on Aging (COAAA), focused on advancing community-engaged research in response to multiple aspects of aging in the community. This presentation will explore the continuum of community-engaged research, to build meaningful collaborations between two entities that shared similar values and identified shared concerns, as demonstrated through three research projects. The projects address three different critical needs among older adults and reflect different levels of engagement, 1) Low-income housing residents, especially older adults with disabilities, face heightened risks during weather-related disasters, 2) The increasing rate of older adult renters that are considered extremely housing cost burdened, and 3) The unique needs of an increasingly diverse population of older adults, in particular the rising number of older adults that are LGBTQ+. The three projects represent different methodological approaches and levels of engagement, illustrating unique funding, staffing, and project implementation activities. Preliminary findings from interviews, focus groups, and survey activities for each research project will be presented. The primary focus will be understanding the development and design of the projects, ranging from consultation, collaboration, to partnership, emphasizing challenges and points of success experienced by AAA and by AFIC.

